# Persistent Heart Rate Variability Deficits Among Dementia Patients With History of Excessive Alcohol Use

**DOI:** 10.1002/alz.090072

**Published:** 2025-01-03

**Authors:** Marianne T Elegores, Shivani Patel, Sarah Elmi

**Affiliations:** ^1^ Ontario Tech University, Oshawa, ON Canada; ^2^ Ontario Tech University, L1G 0C5, ON Canada; ^3^ Ontario Shores Centre for Mental Health Sciences, Whitby, ON Canada; ^4^ University of Toronto, Toronto, ON Canada

## Abstract

**Background:**

Previous literature has highlighted that excessive alcohol use (EAU) is directly linked with permanent neurological damage. Studies have also highlighted gradual improvements in heart rate variability (HRV) after cessation of alcohol use. Moreover, chronic alcohol consumption has also been correlated with reduced HRV and an increase in skin conductance (SC) among healthy adults, leading to a combined decline in cognitive performance. This study examines the persistence of HRV and SC changes among older adults with dementia and a history of alcohol use disorder (AUD).

**Method:**

Participants were recruited from the Geriatric Transitional Unit at Ontario Shores Mental Health Sciences, Whitby, Ontario. Eligibility included a formal diagnosis of dementia and history of lifelong EAU was recorded based on informants. Behavioral and symptoms of dementia was assessed with the Neuropsychiatric Inventory Clinician rating scale. Empatica E4 wristbands was used to record physiological data. Physiological signals such as electrodermal activity (EDA) and heart rate (HR) were recorded. HRV was analyzed using KUBIOS software. T‐tests were used to determine differences in frequency and severity of BPSD, HRV and SC.

**Result:**

28 participants were recruited, 17 male, average age: 73.4, 10 had EAU history (average age 67.8, 2 female). EAU group had a significantly lower root mean square of successive differences (RMSSD) (p = 0.002) and parasympathetic nervous system (PNS) activity (p = 0.021), but significantly higher sympathetic nervous system activity (SNS) (p = 0.007) than participants without EAU. Additionally, the standard deviation of normal beats (SDNN) and RMSSD scores of EAU group were below the normal range. Moreover, EDA was higher in participants without EAU (M = [1.54], SD = [3.46]) compared subjects with EAU (M = [0.43], SD = [0.94]), but the difference was not significant (p = 0.36). There was no statistically significant difference between NPI scores of the 2 groups.

**Conclusion:**

Our findings indicate that alcohol abuse results in long‐term alterations of peripheral nervous system, causing an increase in sympathetic activity and a consequent decrease in parasympathetic activity. We observed that EAU subjects have a diminished ability to vagally mediate heart rate.

